# Mechanisms of ring chromosome formation, ring instability and clinical consequences

**DOI:** 10.1186/1471-2350-12-171

**Published:** 2011-12-21

**Authors:** Roberta S Guilherme, Vera F Ayres Meloni, Chong A Kim, Renata Pellegrino, Sylvia S Takeno, Nancy B Spinner, Laura K Conlin, Denise M Christofolini, Leslie D Kulikowski, Maria I Melaragno

**Affiliations:** 1 Genetics Division, Department of Morphology and Genetics, Federal University of São Paulo, Botucatu Street 740, Zip Code 04023-900, São Paulo, Brazil; 2 Genetics Unit, Instituto da Criança, University of São Paulo, Avenue Dr. Enéas Carvalho de Aguiar 647, Zip Code 05403-000, São Paulo, Brazil; 3 Department of Psychobiology, Federal University of São Paulo, Rua Botucatu, 740, Zip Code 04023-900, São Paulo, Brazil; 4 Division of Human Genetics and Molecular Biology, Children's Hospital of Philadelphia, 34th Street and Civic Center Boulevard, Pa 19104, Philadelphia, Pennsylvania, USA; 5 Gynecology and Obstetricy Division, School of Medicine of ABC, Avenue Príncipe de Gales 821, Zip Code 09060-650, São Paulo, Brazil; 6 Department of Pathology, LIM 03, University of São Paulo, Avenue Dr. Enéas Carvalho de Aguiar 647, Zip Code 05403-000, São Paulo, Brazil

## Abstract

**Background:**

The breakpoints and mechanisms of ring chromosome formation were studied and mapped in 14 patients.

**Methods:**

Several techniques were performed such as genome-wide array, MLPA (Multiplex Ligation-Dependent Probe Amplification) and FISH (Fluorescent *in situ *Hybridization).

**Results:**

The ring chromosomes of patients I to XIV were determined to be, respectively: r(3)(p26.1q29), r(4)(p16.3q35.2), r(10)(p15.3q26.2), r(10)(p15.3q26.13), r(13)(p13q31.1), r(13)(p13q34), r(14)(p13q32.33), r(15)(p13q26.2), r(18)(p11.32q22.2), r(18)(p11.32q21.33), r(18)(p11.21q23), r(22)(p13q13.33), r(22)(p13q13.2), and r(22)(p13q13.2). These rings were found to have been formed by different mechanisms, such as: breaks in both chromosome arms followed by end-to-end reunion (patients IV, VIII, IX, XI, XIII and XIV); a break in one chromosome arm followed by fusion with the subtelomeric region of the other (patients I and II); a break in one chromosome arm followed by fusion with the opposite telomeric region (patients III and X); fusion of two subtelomeric regions (patient VII); and telomere-telomere fusion (patient XII). Thus, the r(14) and one r(22) can be considered complete rings, since there was no loss of relevant genetic material. Two patients (V and VI) with r(13) showed duplication along with terminal deletion of 13q, one of them proved to be inverted, a mechanism known as inv-dup-del. Ring instability was detected by ring loss and secondary aberrations in all but three patients, who presented stable ring chromosomes (II, XIII and XIV).

**Conclusions:**

We concluded that the clinical phenotype of patients with ring chromosomes may be related with different factors, including gene haploinsufficiency, gene duplications and ring instability. Epigenetic factors due to the circular architecture of ring chromosomes must also be considered, since even complete ring chromosomes can result in phenotypic alterations, as observed in our patients with complete r(14) and r(22).

## Background

Ring chromosomes usually result from two terminal breaks in both chromosome arms, followed by fusion of the broken ends, or from the union of one broken chromosome end with the opposite telomere region, leading to the loss of genetic material [1]. Alternatively, they can be formed by fusion of subtelomeric sequences or telomere-telomere fusion with no deletion, resulting in complete ring chromosomes [1-5]. Based on high-resolution molecular karyotyping, other mechanisms of formation of ring chromosomes have been proposed, such as a terminal deletion and a contiguous inverted duplication due to an inv-dup-del rearrangement [6-8]. McGinnis et al [9] analyzed 11 cases of r(21) and found two different mechanisms of ring formation, one resulting from breakage and reunion of the long arms of an intermediate isochromosome or Robertsonian translocation chromosome generating a large dicentric r(21) and the other resulting from breakage in both the short and the long arms of chromosome 21, followed by reunion, duplication and exchange between sister chromatids.

Ring chromosomes have been found for all human chromosomes. Usually the phenotype of ring chromosome patients overlaps that of the deletion of both ends of the respective chromosome syndromes [10]. Nevertheless, the phenotypes associated with ring chromosomes are highly variable, since - in addition to the primary deletion associated with ring formation - secondary loss or gain of material may occur, due to ring chromosome instability [11-15]. In patients with ring chromosomes, sister chromatid exchanges occurring during mitosis usually result in secondary chromosomal abnormalities, such as dicentric rings, interlocked rings, and other structural conformations. These unstable chromosomes can also lead to ring chromosome loss, producing monosomic cells, which may or may not be viable [16-20]. Thus, apart from the deletions due to ring formation, ring instability can also result in other genomic imbalances, with decrease or increase of genetic material and possible consequences on the phenotype.

We report here the mechanisms of ring chromosome formation and ring instability in 14 patients evaluated by cytogenetic and molecular techniques and we discuss their clinical consequences.

## Methods

### Patients

**Figure 1:**
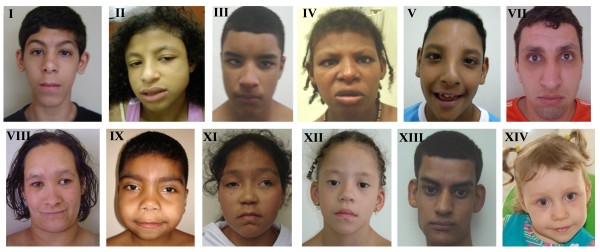
**Patients I-V, VII-IX and XI-XIV, who present ring chromosomes 3, 4, 10, 10, 13, 14, 15, 18, 18, 22, 22 and 22 at ages 16, 12, 14, 22, 8, 23, 28, 7, 11, 7, 24, and 2, respectively**. Patients VI and IX (ring 13 and 18) did not allow picture publication.

We analyzed 14 patients (Figure 1) carrying *de novo *rings derived from chromosomes 3, 4, 10 (two cases), 13 (two cases), 14, 15, 18 (three cases), and 22 (three cases). Patient I, VI and VII were previously reported [20-22]. This study was approved by the ethics committee of the University Federal of São Paulo (CEP 1485-07). Written informed consent was obtained from the patients for publication of this report and accompanying images.

### Clinical Data

**Patient I**. 10 year-old boy, intra uterine growth retardation (IUGR), short stature, triangular face, long and smooth philtrum, retrognathia, large ears, hypoplastic scrotum, cryptorchidism, crossed renal ectopia, moderate development delay and intellectual deficiency. **Patient II**. 11 year-old girl, IUGR, short stature, microcephaly, prominent nasal bridge, pointed and long nose, short philtrum, high palate, dysmorphic ears, hypotonia, neuro-psychomotor delay and intellectual deficiency. **Patient III**. 14 year-old boy, short stature, dolico-trigonocephaly, bilateral epicanthic folds, upslanting palpebral fissures, ocular hypertelorism, left palpebral ptosis, myopia, strabismus, low-set and simplified auricles, broad nasal root, depressed nasal bridge, retrognathism, high-arched palate, long hands, cervical kyphosis, systolic dysfunction with minimal mitral and tricuspid reflux and dilatation of the left ventricle, vesico-uretheral reflux, speech delay and intellectual deficiency, **Patient IV**. 22 year-old girl, short stature, microcephaly, large nasal bridge, long philtrum, dysmorphic ears, esotropia, dermal hypopigmentation regions, semi-flexed legs, Dandy-Walker variant cist, esotropia and mild optic nerve dysplasia at right, ostepenia, ulna shortening, bent legs, hypotonia, neuro-psycho-motor delay and intellectual deficiency. **Patient V**. First genetic evaluation on 3 year-old boy, preterm, IUGR, microcephaly, narrow and oblique forehead, upslanting palpebral fissures, ocular hypertelorism, prominent nasal bridge, high palate, prominent incisors, large and dysmorphic ears, peno-scrotal inversion, scrotal hypoplasia, prominent and large halluces, renal ectopia, hypotonia and severe neuro-psychomotor delay. **Patient VI**. One year-old boy, IUGR, microsomia, microcephaly, micrognathism, bilateral epicanthic folds, long eyelashes, small nose, prominent nasal bridge, long phlitrum, broad helices, low set dysmorphic ears, high palate, thin upper lip, high palate, thoraco-lombar scoliosis, right feet pos-axial polydactyly, hypotonia and neuro-psychomotor development delay. **Patient VII**. 23 year-old male, downslanting palpebral fissures, prominent nose, broad nasal bridge, thin upper lip, upper anus implantation and decreased subcutaneous tissue in gluteal region, hypotrophy of the of the lower limbs, club feet with scar on internal edges from anterior surgical repair, protrusion of the calcaneus, and mild intellectual deficiency. **Patient VIII**. 22 year-old female, short stature, microcephaly, brachycephaly, high forehead, exotropia, hipoplastic alae nasi, hiperextensible knees, rough and drought skin of the lower limbs, generalized hirsutism and mild intellectual disability. **Patient IX**. 7 year-old boy, short stature, microcephaly, brachycephaly, middle face hypoplasia, upper slanting palpebral fissures, ocular hypertelorism, large mouth, downturned angles of mouth, high-arched palate, bifid uvula, peno-scrotal hypospadia, bifid scrotum, bilateral cryptorchidism, bilateral inguinal hernia, vesico-uretheral reflux (grade III), recurrent pulmonary and urinary infections, subclinical hypothyroidism and eczema, hypotonia and moderate neuro-psychomotor development delay. **Patient X**. 5 year-old girl, short stature, esotropia, bilateral epicanthic folds, downturned angles of mouth, large and posterior rotated ears, clinodactyly of 5^th ^fingers, gastro-esophageal reflux and atrial/tricuspid cardiac defects corrected by surgery. **Patient XI**. 11 year-old girl, short stature, ocular hypertelorism, thick eyebrows, esotropia, right palpebral ptosis, bulbous nose, one pre auricular appendix and two pre-auricular pit at right, hepatomegaly and splenomegaly, IgA immunodeficiency, chronic hepatitis, renal tubular acidosis, neuro-psychomotor delay and intellectual deficiency. **Patient XII**. 6 year-old girl, microcephaly, prominent forehead, low set nasal bridge, pectus excavatum, asymmetry the lower limbs, depigmented patches in the upper limbs, minimal ventricular septal defect, mild dysplasia of the tricuspid valve and mild developmental delay. **Patient XIII**. 24 year-old male, hypotonia, elongated and concave face, prominent nose, large and prominent dysmorphic ears, two *café-au-lait *spots, chest asymmetry, dorso-lombar scoliosis, C2-C3 vertebral fusion, neuro-psychomotor delay and intellectual deficiency. **Patient XIV**. Two year-old girl, irregular teeth, large nose, small mouth, a small supernumerary nipple at left, proximal implantation of halluces, hypotonia and mild motor development delay.

### Cytogenetic study

**Figure 2:**
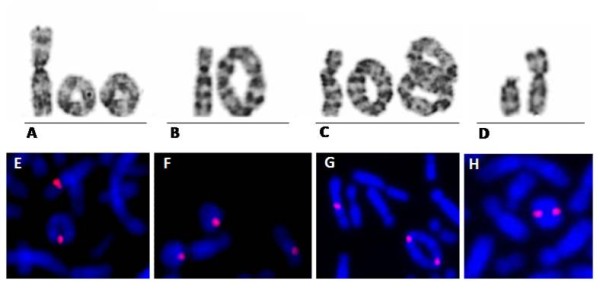
**Examples of ring chromosome instability using G-banding: A) partial metaphase with two ring chromosomes 3; B) a dicentric ring chromosome 10; C) three dicentric ring chromosomes 10, two of them interlocked; D) an open dicentric ring chromosome 15; and by FISH: E) one ring chromosome; F) two ring chromosomes; G and H) dicentric ring**.

**Figure 3:**
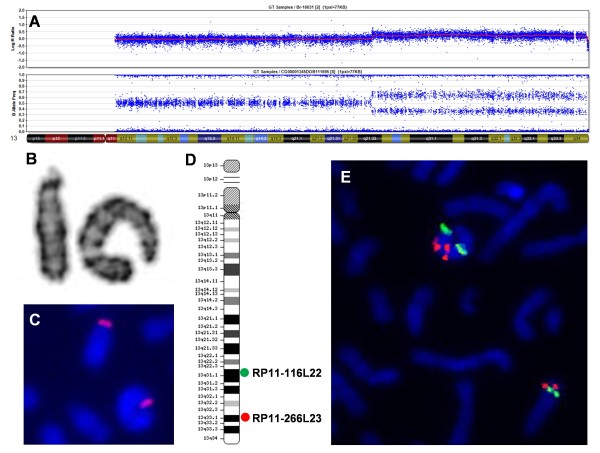
**A) Array result for patient VI showing duplication and deletion of 13q, based on SNP genotyping using the Illumina software**. The upper panel shows the log R ratio, or intensity of each probe. The lower panel shows the B allele frequency, or genotyping results for each single nucleotide polymorphism (SNP). B) Partial karyotype in G-banding showing the r(13) and its normal homologue. C) FISH with pericentromeric probe showing the r(13) and its homologue. D) Ideogram illustrating the chromosome 13 and showing the BACs probes used for FISH. E) FISH-BAC showing the r(13) with duplicated and inverted segment and its normal homologue.

Peripheral blood lymphocytes were obtained from 72-hour cultures and prepared according to standard cytogenetic procedures. Ring instability was verified by counting 300 cells for each patient: 200 after G-banding and 100 using FISH with centromeric or pericentromeric probes (Cytocell, Cambridge, UK) (Figure 2). FISH using pantelomeric probe (Star*FISH Human Chromosome Pantelomeric Probe, Cambio, Cambridge, UK) was performed in cases without terminal deletion, in order to investigate the presence of telomere regions in the ring chromosomes. In patient VI, we also performed FISH with BAC (bacterial artificial chromosomes) probes (RP11-266L23 and RP11-116L22), to determine whether the duplicated region was inverted or not (Figure 3). In patient XIV, FISH was performed using DiGeorge/VCFS-Tuple1 (Cytocell, Cambridge, UK) to confirm the 22q deletion.

### Molecular studies

DNA was isolated from peripheral blood using the Gentra Puregene kit (Qiagen-Sciences, Maryland, USA). The MLPA assay was performed using the *P070 Human telomere 5 probemix kit *(MRC-Hollandâ, Amsterdam, Netherlands) with subtelomeric probes. The MLPA results were analyzed by means of the GeneMarker software. For the array experiments, two different techniques were used: (1) Illumina Quad610 array (Illumina BeadStation, San Diego, CA, USA), performed at the Center for Applied Genomics of the Children's Hospital of Philadelphia according to [23]; (2) for nine patients, the array analysis was also performed at the MolecularCore AFIP laboratory in São Paulo, using the Affymetrix Genome-Wide Human SNP Nsp/Sty 6.0 array (Affymetrix Inc., Santa Clara, CA, USA), followed by standard protocol and as previously described by Guilherme et al [21]. The array data was analyzed using annotation GRCh36/hg18.

## Results

Table 1 show that the ring loss frequency in the different patients varied from 1.7% to 12.7% in metaphase cells, and of secondary aberrations (duplicated ring, two rings, interlocked rings or open ring) from 0.3% to 6.3%. The total of cells with chromosome instability varied from 4.0% to 16.3%. Table 2 shows the cytogenetic and molecular results obtained for the patients and the deleted regions of the ring chromosomes. Patient XIV presented, in addition to the terminal deletion in 22q, a 22q11.2 deletion in the ring chromosome, confirmed by FISH.

**Table 1:** Distribution of metaphases obtained from 72-hour cultures of peripheral blood lymphocytes, according to the number of chromosomes and the presence of secondary aberrations derived from the ring chromosomes.

Patients Metaphases	I r(3)	II r(4)	III r(10)	IV r(10)	V r(13)	VI r(13)	VII r(14)	VIII r(15)	IX r(18)	X r(18)	XI r(18)	XII r(22)	XIII r(22)	XIV r(22)
46 chromosomes with a ring	262	287	251	253	271	264	260	277	268	275	272	276	288	285
45 chromosomes without ring	19	5	38	33	12	19	35	11	17	17	20	15	11	13
Secondary aberrations	19	8	11	14	17	17	5	12	15	8	8	9	1	2

Total	300	300	300	300	300	300	300	300	300	300	300	300	300	300

Percentage of cells with secondary aberrations and without the ring (%)	12.7	4.3	16.3	15.7	9.6	12.0	13.3	7.7	10.7	8.3	9.3	8.0	4.0	5.0

**Table 2:** Cytogenetic and molecular findings in patients with ring chromosomes.

Patient	Karyotype	Genomic imbalance	Subtel MLPA	Pantel FISH
I	46,XY,r(3)(p26.1q29).arr 3p26.3p26.1(307,820-6,045,520) ×1	del 3p: 5.7 Mb	3p- 3q+	-
II	46,XX,r(4)(p16.3q35.2).arr 4p16.3(449,000-1,827,772)×1	del 4p: 1.3 Mb	4p- 4q+	-
III	46,XY,r(10)(p15.3q26.2).arr 10q26.2q26.3(127,589,040-135,056,940)×1	del 10q: 7.4 Mb	10p+ 10q-	+
IV	46,XX,r(10)(p15.3q26.13).arr 10p15.3(158,900-979,500)× 1, 10q26.13q26.3 (126,660,267-135,374,720)× 1	del 10p: 820 Kb del 10q: 8.6 Mb	10p- 10q-	
V	46,XY,r(13)(p13q33.1).arr 13q33.1(101,543,509-103, 001,462)×3,13q33.1q34(103,003,268-114,142,980)×1	dup 13q: 1.5 Mb del 13q: 11.1 Mb	"13p"+ 13q-	
VI	46,XY,r(13)(p13q34).arr 13q21.33q34(70,141,036-113, 656,958)×3,13q34(113,759,040-114,123,122)×1	dup 13q:43.5Mb del 13q: 364 Kb	"13p"+ 13q-	
VII	46,XY,r(14) (p13q32.33).arr (14)×2	no deletion	"14p"+ 14q+	-
VIII	46,XX,r(15)(p13q26.2).arr 15q26.2q26.3(94,810,000-100,338,900)×1	del 15q: 5.5 Mb	"15p"+ 15q-	
IX	46,XY,r(18)(p11.32q22.2).arr 18p11.32p(20-1,377,020)× 1,18q22.2q23(65,015,880-75,849,120)×1	del 18p: 1.3 Mb del 18q: 11.1 Mb	18p- 18q-	
X	46,XX,r(18)(p11.32q21.33).arr 18q21.33q23(57,446,374-76,117,140)×1	del 18q: 18.6 Mb	18p+ 18q-	+
XI	46,XX,r(18)(p11.21q23).arr 18p11.32p11.21(178,680-15, 545,050)×1,18q23(74,427,840-76,027,800)×1	del 18p: 15.3 Mb del 18q: 1.7 Mb	18p- 18q-	
XII	46,XX,r(22)(p13q13.33).arr (22)×2	no deletion	"22p"+ 22q+	+
XIII	46,XY,r(22)(p13q13.2).arr 22q13.2q13.33(42,437,109-49,562,479)×1	del 22q: 7.1 Mb	"22p"+ 22q-	
XIV	46,XX,r(22**)(**p13q13.2).arr 22q11.21(17,257,787-18,710, 895)×1,22q13.2q13.33(41,744,274-49,562,479)×1	del 22q: 1.4 Mb del 22q: 7.8 Mb	"22p"+ 22q-	

Subtel MLPA: MLPA with subtelomeric probes; Pantel FISH: FISH signals on ring chromosomes obtained with pantelomeric probes; (-): absent; (+): present; (" "): for acrocentric chromosomes, probes are located proximally on their long arms.

The SNP array and FISH results obtained for patient VI who has an r(13) revealed a 43.5 Mb inverted duplication at 13q21.33q34 followed by a 364 kb terminal deletion at 13q34 (Figure 3).

In the patients reported here, different mechanisms of ring formation were found: breaks in both chromosome arms followed by end-to-end reunion (patients IV, VIII, IX, XI, XIII and XIV), a break in one chromosome arm followed by fusion with the subtelomeric region of the other (patients I and II), a break in one chromosome arm followed by fusion with the opposite telomeric region (patients III and X), fusion of two subtelomeric regions (patient VII), and telomere-telomere fusion (patient XII). Thus, the r(14) and one r(22) can be considered complete rings, since there was no loss of relevant genetic material. A more complex mechanism of formation was found in both patients with r(13), with a terminal deletion but also with a contiguous duplication. In patient VI, the duplicated 43.5 Mb segment next to the breakpoint at 13q34 was inverted. In patient V, it was not possible to confirm if the duplicated segment was also inverted.

## Discussion

In this work, the array and FISH techniques allowed determination of the breakpoints and genomic unbalances and also definition of the mechanism of formation of the ring chromosomes. We identified several ring chromosome formation mechanisms, including rings with deletion in one or both arms, complete rings, and rings formed by a complex mechanism, due to inv-dup-del in two patients with ring chromosomes 13. The MLPA assay allowed evaluating the presence of subtelomeric regions in the ring chromosomes.

According to Kosztolányi [24], a ring chromosome is considered unstable when secondary aberrations were found in more than 5% of the mitoses counted. Thus, most of our patients showed unstable ring chromosomes, except patient II who presented a ring 4, and patients XIII and XIV who presented rings 22, revealing no clear correlation between size and ring instability. Our data are in accordance with Kistenmacher and Punnet [25], who stated that behavioral and structural instability of a ring is a function of its genetic content rather than its initial size. The most important factor affecting the phenotype of patients with ring chromosome is the chromosome involved in the rearrangement and the extension of the deletion of genome segments that contain crucial genes for a normal development. Thus, each patient will present their own phenotypic features considering the genes deleted from one or both chromosome arms. We observed that none of the ring chromosomes described in the patients from our sample has similar breakpoints, same those are formed by the same chromosome. Probably there is no specific hotpoint in the chromosomes more favorable to these breaks and reunion, that resulting in the ring. Some characteristics, such as delay growth, are usually associated with any autosome ring chromosome, possibly due to ring instability [19,26]. But in patients with r(15) the growth delay is more severe and evident when *IGF1R *(*insulin-like growth factor 1 receptor precursor*) gene, located in 15q26.3, is loss, as observed in our patient with r(15). Similarly, our patient with r(4) presented severe intrauterine growth delay, a feature usually found in the del (4p) syndrome but not in the del (4q) syndrome. Thus, stature is not related just with the instability of the ring, but also correlates with the haploinsufficiency of stature related genes.

Another factor that could influence the phenotype is the configuration of the ring chromosome that could change the gene expression and cause clinical abnormalities [15,27,28], as observed in our patients VII and XII who presented complete rings without deletion. Another interesting observation is that patients III and × present certain features associated to genes not deleted in the ring chromosomes. Patient III with r(10) presents bilateral cryptorchidism and vesico-uretheral reflux although he has no deletion of *RET, PAX2*, FGFR2, *GFRA1 *and *EMX2 *genes mapped in 10q, postulated as candidate genes for urinary and/or genital development [29]. Also, our patient with r(14) presented seizures and hypopigmented area in posterior pole in both eyes, which are features commonly found in individuals with a ring 14 and have been attributed to genes proximally located on 14q11q13 and q32.2, respectively [15,21,30]. Castermans et al [31] reported a patient with autism and coloboma, in which the ring formation was associated with silencing of the *AMISYN *gene, located near the breakpoint, also suggesting that the position effect can have clinical consequences possibly due to gene silencing.

From the recent discovery that ring chromosomes can present duplicated genomic segments [7,8], phenotypic correlation in ring patients cannot be done by assuming a simple deletion without excluding the detection of additional duplicated segments. Terminal deletions were found to be associated to duplications near the breakpoint in our patients V and VI who present not only the features found in deletion 13 and r(13) syndromes but a variable clinical picture related to the size of the duplication. Thus, this mechanism inv-dup-del of ring formation has important implications for the phenotype, since these ring chromosomes result not only in partial monosomy but also in partial trisomy.

## Conclusions

In light of all these findings, we concluded that the large spectrum of symptoms and their severity in patients with ring chromosomes can be attributed to different factors. Besides the deletion in one or in both chromosome arms occurring in ring formation, the resultant secondary genetic imbalance due to ring instability and epigenetic factors need also to be taken into account in the evaluation of the genetic consequences and in the attempt to reach a better understanding of the genotype-phenotype correlations.

## Competing interests

The authors declare that they have no competing interests.

## Authors' contributions

RSG performed cytogenetic and FISH studies and wrote the paper. VFAM and CAK carried out the data clinical of the patients. RP performed SNP array using the Affymetrix platform and analyzed the results. SST performed cytogenetic studies. NBS and LKC performed SNP array using the Illumina platform and analyzed the results. DMC performed MLPA study and analyzed the results. LDK and MIM coordinated the study and helped to draft the manuscript. All authors read and approved the final manuscript.

## Pre-publication history

The pre-publication history for this paper can be accessed here:

http://www.biomedcentral.com/1471-2350/12/171/prepub
